# Supplementary material for: Aberrant preparation of hand movement in schizophrenia spectrum disorder: an fMRI study
Source: Brain Commun. 2025 Apr 25;7(2):fcaf148. doi: 10.1093/braincomms/fcaf148 (PMC12022610; doi:10.1093/braincomms/fcaf148)
Supplement: fcaf148_Supplementary_Data [file fcaf148_supplementary_data.pdf]

Supplementary Material

Supplementary Table 1: Correlation between neural activation (cluster's eigenvariates) and symptom score in SSD patients

Cluster consisting mainly	symptom	Pearson correlation coeeficient: r	p	Effect size (Fisher's z)	Spearman correlation coeeficient: rho	p	Effect size (Fisher's z)
Left insula and putamen<br><br>preparation for active movement with own hand feedback	SAPS_I	-0.076	0.752	-0.076	-0.281	0.230	-0.289
	SAPS_14	-0.614	**0.004**	-0.716	-0.585	0.007	-0.669
	SAPS_15	-0.473	**0.035**	-0.514	-0.466	0.039	-0.504
	residual positive symptoms	-0.341	0.142	-0.355	-0.278	0.241	-0.282
	SANS_I	-0.076	0.752	-0.076	-0.281	0.230	-0.289
	SANS_II	-0.241	0.305	-0.246	-0.282	0.228	-0.290
	SANS_III	-0.580	**0.007**	-0.663	-0.608	0.004	-0.706
	SANS_IV	-0.494	**0.027**	-0.542	-0.510	0.022	-0.563
	SANS_V	0.069	0.774	0.069	0.0.099	0.877	0.100
Left middle temporal gyrus<br><br>preparation for passive movement with own hand feedback	SAPS_I	-0.495	**0.027**	-0.542	-0.31	0.183	-0.321
	SAPS_14	-0.087	0.716	-0.087	-0.155	0.515	-0.156
	SAPS_15	-0.468	**0.037**	-0.508	-0.307	0.187	-0.318
	residual positive symptoms	-0.162	0.496	-0.163	-0.090	0.707	-0.090
	SANS_I	-0.495	**0.027**	-0.542	-0.310	0.183	-0.321
	SANS_II	0.046	0.847	0.046	-0.117	0.624	-0.117
	SANS_III	-0.270	0.250	-0.277	-0.151	0.526	-0.152
	SANS_IV	0.029	0.905	0.029	-0.0008	0.997	-0.0008
	SANS_V	0.235	0.319	0.239	0.259	0.271	0.265
Right angular gyrus<br><br>preparation for passive movement with own hand feedback	SAPS_I	-0.448	**0.047**	-0.483	-0.502	0.024	-0.551
	SAPS_14	0.059	0.804	0.059	0.079	0.742	0.079
	SAPS_15	-0.402	0.079	-0.426	-0.085	0.720	-0.086
	residual positive symptoms	-0.066	0.783	-0.066	-0.029	0.903	-0.029
	SANS_I	-0.448	**0.047**	-0.483	-0.502	0.024	-0.551
	SANS_II	-0.118	0.620	-0.119	-0.222	0.348	-0.225
	SANS_III	-0.255	0.278	-0.261	-0.089	0.710	-0.089

	SANS_IV	-0.203	0.391	-0.206	-0.241	0.307	-0.246
	SANS_V	0.257	0.274	0.263	0.298	0.201	0.308
Lobule VIII of right cerebellar hemisphere	SAPS_1	0.038	0.874	0.038	-0.177	0.456-	-0.179
	SAPS_14	-0.616	**0.004**	-0.719	-0.609	0.004	-0.707
preparation for active movement with own hand feedback	SAPS_15	-0.300	0.198	-0.310	-0.492	-0.028	-0.538
	residual positive symptoms	-0.447	**0.048**	-0.480	-0.116	0.627	-0.116
	SANS_I	0.038	0.874	0.038	-0.177	0.458	-0.179
	SANS_II	-0.287	0.220	-0.295	-0.417	0.067	-0.444
	SANS_III	-0.453	**0.045**	-0.488	-0.491	0.028	-0.537
	SANS_IV	-0.374	0.105	-0.393	-0.485	0.030	-0.529
	SANS_V	-0.599	**0.005**	-0.691	-0.378	0.100	-0.398

Note: SAPS scale for the assessment of positive symptoms, SAPS_I hallucinations, SAPS_II delusions, SAPS_14 delusions of reference, SAPS_15 delusions of being controlled, SAPS_III bizarre behavior, SAPS_IV positive formal thought disorder, SAPS_res residual positive symptom (SAPS_III + SAPS_IV + SAPS_V). SPQ-B: schizotypal personality questionnaire-brief, SPQ-B_cogn_perc SPQ-B cognitive perceptual deficit, SPQ_Interpers SPQ interpersonal deficit, SPQ_desorg SPQ-B disorganization, SPQ_total SPQ-B total score accumulated from SPQ_cogn_perc + SPQ_interpers + SPQ_desorg, SANS scale for the assessment of negative symptoms, SANS_I affective flattening or blunting, SANS_II alogia, SANS_III avolition/apathy, SANS_IV anhedonia/asociality, SANS_V attention, SANS_summe_global sum of item 22 to 24, SANS_Gesamtkalenwert sum of SANS_I to SANS_V. **Bold** values represent significant differences between HC and SSD patients ($p < 0.05$, uncorrected).

Supplementary Table 2: Partial correlation (partialling out SANS score) between neural activation (cluster's eigenvariates) and symptom score in SSD patients

Cluster consisting mainly	symptom	Pearson correlation coeeficient: r	p	Effect size (Fisher's z)	Spearman correlation coeeficient: rho	p	Effect size (Fisher's z)
Left insula and putamen<br><br>preparation for active movement with own hand feedback	SAPS_I	0.258	0.286	0.264	0.011	0.964	0.011
	SAPS_14	-0.471	**0.042**	-0.512	-0.414	0.078	-0.440
	SAPS_15	-0.321	0.195	-0332	-0.348	0.158	-0.364
	residual positive symptoms	-0.245	0.312	-0.250	-0.222	0.361	-0.226
Left middle temporal gyrus<br><br>preparation for passive movement with own hand feedback	SAPS_I	-0.475	**0.040**	-0.517	-0.291	0.226	-0.300
	SAPS_14	0.046	0.850	0.046	-0.094	0.702	-0.094
	SAPS_15	-0.516	**0.028**	-0.571	-0.436	0.071	-0.467
	residual positive symptoms	-0.112	0.648	-0.113	-0.069	0.790	-0.069
Right angular gyrus<br><br>preparation for passive movement with own hand feedback	SAPS_I	-0.332	0.165	-0.345	-0.374	0.115	-0.393
	SAPS_14	0.364	0.125	0.382	0.430	0.066	0.460
	SAPS_15	-0.295	0.235	-0.304	0.251	0.314	0.257
	Sa_SAPS_15	-0.520	**0.027**	-0.570	-0.368	0.133	-0.386
	residual positive symptoms	0.034	0.890	0.034	0.039	0.873	0.039
Lobule VIII of right cerebellar hemisphere<br><br>preparation for active movement with own hand feedback	SAPS_I	0.384	0.107	0.405	0.045	0.855	0.045
	SAPS_14	-0.489	**0.033**	-0.535	-0.520	0.020	-0.588
	SAPS_15	0.052	0.839	0.052	-0.435	0.071	-0.466
	residual positive symptoms	-0.232	0.340	-0.236	0.113	0.646	0.113

Note: SAPS scale for the assessment of positive symptoms, SAPS_I hallucinations, SAPS_II delusions, SAPS_14 delusions of reference, SAPS_15 delusions of being controlled, SAPS_III bizarre behavior, SAPS_IV positive formal thought disorder, SAPS_res residual positive symptom (SAPS_III + SAPS_IV + SAPS_V). SPQ-B: schizotypal personality questionnaire-brief, SPQ-B_cogn_perc SPQ-B cognitive perceptual deficit, SPQ_Interpers SPQ interpersonal deficit, SPQ_desorg SPQ-B disorganization, SPQ_total SPQ-B total score accumulated from SPQ_cogn_perc + SPQ_interpers + SPQ_desorg, SANS scale for the assessment of negative symptoms, SANS_I affective flattening or blunting, SANS_II alogia, SANS_III avolition/apathy, SANS_IV anhedonia/asociality, SANS_V attention, SANS_summe_global sum of item 22 to 24, SANS_Gesamtkalenwert sum of SANS_I to SANS_V. Bold values represent significant differences between HC and SSD patients ($p < 0.05$, uncorrected
